# Health-related quality of life in parents of school-age children with Asperger syndrome or high-functioning autism

**DOI:** 10.1186/1477-7525-4-1

**Published:** 2006-01-04

**Authors:** Hiie Allik, Jan-Olov Larsson, Hans Smedje

**Affiliations:** 1 Karolinska Institutet, Dept. of Woman and Child Health, Child and Adolescent Psychiatric Unit, Astrid Lindgren Children's Hospital, SE-171 76 Stockholm, Sweden; 2 Uppsala University, Dept. of Neuroscience, Child and Adolescent Psychiatric Unit, SE-751 85 Uppsala, Sweden

## Abstract

**Background:**

The estimated prevalence rate of Pervasive Developmental Disorders (PDD) in children is 6 per 1.000. Parenting children who are intellectually impaired and have PDDs is known to be linked to the impaired well-being of the parents themselves. However, there is still little available data on health-related quality of life (HRQL) in parents of children with Asperger Syndrome (AS) and High-Functioning Autism (HFA), or other PDD diagnoses in children of normal intelligence. The present study aimed to evaluate aspects of HRQL in parents of school-age children with AS/HFA and the correlates with child behaviour characteristics.

**Methods:**

The sample consisted of 31 mothers and 30 fathers of 32 children with AS/HFA and 30 mothers and 29 fathers of 32 age and gender matched children with typical development. Parental HRQL was surveyed by the use of the 12 Item Short Form Health Survey (SF-12) which measures physical and mental well-being. The child behaviour characteristics were assessed using the structured questionnaires: The High-Functioning Autism Spectrum Screening Questionnaire (ASSQ) and The Strengths and Difficulties Questionnaire (SDQ).

**Results:**

The mothers of children with AS/HFA had lower SF-12 scores than the controls, indicating poorer physical health. The mothers of children with AS/HFA also had lower physical SF-12 scores compared to the fathers. In the AS/HFA group, maternal health was related to behaviour problems such as hyperactivity and conduct problems in the child.

**Conclusion:**

Mothers but not fathers of children with AS/HFA reported impaired HRQL, and there was a relationship between maternal well-being and child behaviour characteristics.

## Background

The prevalence of Pervasive Developmental Disorders (PDD) in children has increased from 0.4 in 1.000 during the 1970s to current estimates of up to 6 per 1.000. This increase is presumably a consequence of improved ascertainment and considerable broadening of the diagnostic concept [1]. While PDDs were previously only diagnosed in children with mental retardation, recent studies suggest that approximately 50% of individuals diagnosed with PDDs have normal intelligence [2], and a minimum prevalence of 2 out of every 1.000 for PDDs in mainstream school children was reported in a recent study [3]. Asperger syndrome (AS) and high-functioning autism (HFA) are PDD diagnoses in individuals of normal intelligence [4] characterized by pervasive impairment in several areas of development: reciprocal social interaction skills, communication skills, and the presence of stereotyped behaviour, interests, or activities. AS is distinguished from HFA primarily by a lack of clinically significant language delay [5]. The majority of children with AS or HFA live in families along with their parents. Caregiving of a child with a PDD may be associated with high levels of distress and burden [2] which potentially undermine the mental and physical health of the parents of these children. While there is much data available about parenting children with PDDs and associated intellectual impairment, only a few studies have explored the health-related quality of life (HRQL) in parents of children with AS or HFA [6].

Parenting children with developmental disabilities, among them PDDs with intellectual disability, is associated with impaired mental health [7,8], higher levels of stress [8-11], sense of devaluation and blame [9], and also impaired physical functioning, tiredness or exhaustion in mothers and fathers [12,13]. For example, Weiss [7] reported that many parents of children with PDDs experienced feelings of intense anger, guilt, depression or anxiety most of the time. Moreover, these feelings were frequently expressed in psychosomatic problems.

Due to the scarcity of data about the HRQL of parents of children of normal intelligence with PDDs, we deem it relevant to also take into account research on the well-being of parents of children with other types of disorders, such as developmental disabilities or severe mental health problems. Using data from the Wisconsin Longitudinal Study, Seltzer et al. [14] explored parental attainment and well-being at mid-life in parents of children with developmental disabilities and parents of children with severe mental health problems. The parents of children with developmental disabilities accommodated to their child's needs early on, for example, by restricting their social life and making changes in family routines. However, parents of children with severe mental health problem were not as accommodating. At a follow-up, the physical and mental health of parents of children with developmental disabilities did not differ from that of a normative group, while parents of children with severe mental health problems displayed poorer physical health and elevated levels of depressive symptoms. Similarly, Magana et al. [15] also found higher rates of physical health problems in mothers caring for their adult children with mental illness. Notably, neither Seltzer et al. nor Magana et al. stated that individuals with PDDs were included in their studies.

Comparisons between mothers and fathers of a child with a developmental disability have displayed different [12,16,17] as well as similar [11] levels of perceived stress and impaired health. A Swedish report about parents of children with Down's syndrome [12] indicated that mothers had lower scores of self-perceived vitality, and also that they spent more time caring for their child than the fathers. Moreover, a recent family study by Little [6], including children with AS, reported that mothers experienced more stress and pessimism about the child's future, and used antidepressants or other therapy more frequently than the fathers. In the same report, mothers of children with AS found coping strategies such as communication and consultation with family, friends, and professionals more helpful than the fathers did.

Parental stress and health outcome is related to child characteristics such as the severity of the core disability or main diagnosis, the age of the child, and the extent of coexisting behaviour problems [18,19]. It has been suggested that such coexisting behaviour problems in the child predict parental stress to a higher extent than the severity of the intellectual or adaptive functioning [19]. Notably, coexisting behaviour or psychiatric problems are common in individuals with AS or HFA [20-22].

The present study which is a part of a longitudinal investigation of school-age children with AS/HFA and their families [23] focused on the HRQL in parents of children with AS/HFA. More specifically, the aims were to explore: 1) whether the raising of a child with AS/HFA is associated with impaired parental HRQL; 2) if there are differences in the HRQL between mothers and fathers in families with a child diagnosed with AS/HFA; and 3) whether parents' health within the AS/HFA group is related to child behaviour characteristics.

## Methods

### Participants

#### The AS/HFA group

The AS/HFA group consisted of 31 mothers (mean age 42.4, 28–54 yrs) and 30 fathers (mean age 45.6, 35–64 yrs) of 32 children with AS/HFA (mean age 10.8, 8–12 yrs). Our study sample was selected from a total of 122 children with a clinical diagnosis of AS, who were registered at three PDD-habilitation centres in Stockholm. Since another aim of our research project was to elucidate whether sleep patterns of school-age children of normal intelligence and PDD differ from sleep patterns of typically developing children, the following exclusion criteria were employed: suspected mental retardation, essential language delay, the presence of physical disabilities, seizure disorders, and ongoing medication: factors known to affect sleep in children [24,25]. Thirty-two of these 122 children were included in our study sample. The reasons for non-inclusion were as follows: 37 families were unwilling to participate; 9 children had physical disabilities or seizure disorders; 35 children were receiving ongoing medication, and 9 children had mental retardation or a history of essential language delay. Before entering our study, the 32 participating children were also subjected to a diagnostic reassessment based on the ICD-10 research criteria [26], performed by the first author of this study. The diagnostic reassessment revealed that 13 children fulfilled ICD-10 criteria for autistic disorder, and 19 fulfilled ICD-10 criteria for AS. Moreover, with respect to school situation, 13 children attended regular classes in mainstream schools; 4 of these 13 children received extra support from school assistants, and 19 children attended classes or schools for children with various special needs. Further details of the sampling procedure and of the diagnostic reassessment of the PDD sample has been presented in detail elsewhere [23].

#### The control group

The control group consisted of 30 mothers (mean age 40.3, 31–51 yrs) and 29 fathers (mean age 42.7, 35–53 yrs) to 32 typically developing children (mean age 10.9, 8–13 yrs). The 28 boys and 4 girls of the control group who were recruited via school nurses were included if they: 1) were of the same age and gender as the children with AS/HFA; 2) resided in the same local communities as children with AS/HFA and attended regular classes in mainstream schools; 3) had no mental, developmental, or physical disabilities according to school medical records; and 4) were not receiving ongoing prescription medication.

There were no statistically significant differences regarding sociodemographic factors between parents of the AS/HFA and control groups (Table 1).

**Table 1:** Demographic data for the participants in the Asperger syndrome (AS)/high-functioning autism (HFA) and control groups

	*AS/HFA group N (%)*	*Control group N (%)*
Family status		
*nuclear*	21 (65.6)	28 (87.5)
*single parent*	5 (15.6)	2 (6.2)
*one step-parent*	6 (18.7)	2 (6.2)
		
High-school education		
*mothers*	20/31 (64.5)	19/30 (63.3)
*fathers*	20/30 (66.6)	17/29 (58.6)
		
Gainful employment of parents		
*mothers*	23/31 (74.1)	28/30 (93.3)
*fathers*	28/30 (93.3)	28/29 (96.5)
On sick leave (for any illnesses)		
		
*mothers*	3/31 (9.6)	1/30 (3.3)
*fathers*	0/30	1/29 (3.4)
		
Age of parents (years)		
*mothers*	42.4 ± 6.7	40.3 ± 5.1
*fathers*	45.6 ± 6.9	42.7 ± 4.9

*Fischer's Exact test *or *Mann-Whitney test *(age of parents). All differences between parents of the two groups were statistically non-significant.

### Procedure

On receipt of written consent from all participants, the first author visited each family (n = 64) at home. Data for the current analysis was collected simultaneously with data for a study of children's sleep patterns, described elsewhere [23]. The instruments used to assess parental HRQL and the childen's behaviour were distributed to the families at the first home visit. Parents were asked to convey the teacher questionnaires to their child's teacher, and teachers subsequently mailed their completed forms to the first author. Each parent separately filled in the HRQL instrument. The questionnaires were returned to the first author via a second home visit, a parental visit to the clinic, or by mail.

The study was approved by the Ethical Committee at the Karolinska Hospital, Stockholm, Sweden.

### Measures

#### Parental HRQL

The 12 Item Short-Form Health Survey (SF-12), a validated 12 item questionnaire was used to measure parental HRQL [27,28]. The SF-12 generates two scores, the Physical Component Summary (PCS-12), and the Mental Component Summary (MCS-12) score. The SF-12 has previously been used to measure the well-being of caregivers for relatives suffering from different chronic medical conditions [29,30]. In the current report, parental SF-12 scores were compared to Swedish population means [31]. In addition, questions about sociodemographics were added to the SF-12.

#### Child behaviour characteristics

The High-Functioning Autism Spectrum Screening Questionnaire (ASSQ), a 27 item checklist, was included as a measure of autism-related symptoms [32]. Eleven items cover impairments in social interaction, five restricted and repetitive behaviour, six communication problems, and five motor clumsiness and associated symptoms. Both parent and teacher ASSQ versions have shown satisfactory test-retest reliability, inter-rater reliability, and validity [32]. ASSQ data for children of the AS/HFA and control groups are presented in our previous report [23]. Briefly, the mean parental and teacher ASSQ scores for children in the AS/HFA group were 21.2 (*SD *= 8.7) and 21.0 (*SD *= 10.1), versus 0.8 (*SD *= 1.7) and 1.7 (*SD *= 2.3) for children in the control group (*p *< 0.0001, *t*-test for paired samples).

The Strengths and Difficulties Questionnaire (SDQ) was included as a measure of aspects of social competence and psychopathology of the child. The SDQ comprises 25 items, distributed on 5 subscales of 5 items each: the prosocial behaviour subscale (a measure of the child's ability to be considerate, to share, to be helpful and to be kind to younger children); the hyperactivity subscale; the emotional symptoms subscale; the conduct problems subscale and the peer problems subscale [33]. The psychometric properties of the Swedish version of the SDQ have been described as satisfactory elsewhere [34,35]. The current study used both parent and teacher SDQ versions, and ratings showed that children in the AS/HFA group revealed statistically significant higher scores on all subscales, except the prosocial behaviour subscale, where the opposite was the case.

### Statistical analyses

#### Comparisons between the AS/HFA and the control groups

Parental PCS-12 and MCS-12 scores were compared between the AS/HFA and control groups using linear regression, while controlling for parental and child's ages. Difference in HRQL between mothers and fathers in the AS/HFA group was compared to the HRQL difference in the control group, using linear regression, and controlling for parental and child's ages. To calculate the PCS-12 or the MCS-12 score difference between mothers and fathers: mothers' PCS-12 (MCS-12) score was subtracted from fathers' PCS-12 (MCS-12) score.

#### Analyses within the AS/HFA group

The association between parental HRQL and child behaviour characteristics, ASSQ and SDQ scores, within the AS/HFA group was explored using linear regression, while controlling for parental age, age and gender of the child [11,13,36]. When analyzing the relationship between paternal HRQL and child behaviour characteristics, an additional factor was taken into consideration, namely, if the father lived together with a child (yes – is living together, no – is not living together with a child). Since parent and teacher SDQ conduct problems scores had skewed distributions, the logarithmic values were used.

Our HRQL and SDQ data had discrete, bounded and skewed distributions. Therefore, in addition to parametric analyses, non-parametric bootstrap methods were run in Stata [37]. Results of these non-parametric analyses (data not presented here) were similar to the results obtained by the conventional parametric analyses. Our findings with regard to the similarity between the results obtained by parametric and non-parametric methods, coincide with suggestions from previous research [38].

Sociodemographic data were compared by using the Fisher's Exact test (categorical variables) and the Mann-Whitney test (parental age). *T*-test for paired samples was used to compare ASSQ and SDQ scores between children in the AS/HFA and control groups. The Statistical Package for Social Sciences (SPSS) [39] and Stata [37] were used. Significance level *p *< .05 was regarded as statistically significant.

## Results

### Comparisons between the AS/HFA and the control groups

#### Maternal HRQL

Mothers in the AS/HFA group reported lower PCS-12 score, i.e. poorer physical health, than mothers in the control group (44.7 versus 52.5), while controlling for mothers' and child's ages (Table 2). The PCS-12 Swedish norm for 40–44-year-old females is 51.2 [31]. Thus, the score for the control group in the current report resembles data from the norm population mean, while the score for the AS/HFA group is lower than the norm population mean.

**Table 2:** Physical (PCS-12) and Mental Component Summary (MCS-12) scores and PCS-12/MCS-12 differences between mothers and fathers of the AS/HFA and control groups

*SF-12 score*	*AS/HFA group Mean (SD)*	*n*	*Control group Mean (SD)*	*n*	*β*	*SE*	*p*	*95% CI*	
1. Mothers' PCS-12	44.7 (10.8)	31	52.5 (7.4)	30	-8.5	2.4	.001	-13.3	-3.6
2. Mothers' MCS-12	49.1 (11.1)	31	52.0 (9.6)	30	-2.7	2.7	.32	-8.2	2.7
3. Fathers' PCS-12	49.8 (6.9)	30	53.0 (6.8)	29	-2.1	1.8	.24	-5.7	1.5
4. Fathers' MCS-12	51.3 (7.8)	30	53.6 (6.1)	29	-2.7	1.9	.16	-6.5	1.1

5. PCS-12 difference	4.7 (13.8)	29	-0.3 (9.1)	29	6.9	3.1	.03	0.6	13.2
6. MCS-12 difference	2.8 (11.7)	29	0.5 (11.0)	29	1.5	3.1	.64	-4.8	7.7

Each row is a separate Linear regression with the SF-12 score as the dependent variable. The independent variables were: group (AS/HFA vs. control), parental age, and child's age. Parental differences in the SF-12 scores (Items 5 and 6) were calculated as following: Fathers' PCS-12 (MCS-12) minus Mothers' PCS-12 (MCS-12). Positive value indicates better health for the father.

The MCS-12 score, reflecting the mental health status, did not differ between mothers of the AS/HFA and control groups (49.1 versus 52.0). Notably, the MCS-12 Swedish norm for 40–44-year-old females is 52.4 [31].

#### Paternal HRQL

Neither PCS-12 (49.8 versus 53.0) nor MCS-12 scores (51.3 versus 53.6) differed between fathers of the AS/HFA and control groups, while controlling for fathers' and child's ages (Table 2). The PCS-12 Swedish norm for 40–44-year-old males is 51.4 and the MCS-12 norm is 53.8 [31].

#### Differences in HRQL between mothers and fathers

The PCS-12 score difference between mothers and fathers among the parents in the AS/HFA group was statistically significantly greater than the difference among the parents in the control group (Table 2). Thus, the mothers in the AS/HFA group reported poorer physical health status than the fathers. The MCS-12 score difference between mothers and fathers was similar between parents in the AS/HFA group and parents in the control groups.

### The association between parental HRQL and child behaviour characteristics in the AS/HFA group

Parental HRQL was not related to the parent or teacher-rated ASSQ scores of the child. Further, there were significant relationships between maternal HRQL and SDQ scores of the child (Table 3). Higher PCS-12 score – indicating better physical health of the mother – was related to a higher teacher-rated prosocial behaviour score, i.e. better social competence of the child. Further, a higher MCS-12 score – indicating better mental health of the mother – was related to higher scores of parent-rated prosocial behaviour, and lower scores of parent-rated hyperactivity and conduct problems in the child. There was no association between paternal MCS-12/PCS-12 scores and SDQ scores of the child.

**Table 3:** Relationships between mothers' Physical (PCS-12) and Mental Component Summary (MCS-12) scores and the teacher- or parent-rated SDQ scores of the child within the AS/HFA group

*Relationship*	*β*	*SE*	*z*	*p*	*95% CI*	
1. **Mothers' PCS-12 **teacher SDQ **prosocial**	1.8	0.8	2.1	0.03	0.11	3.62
2. **Mothers' MCS-12 **parent SDQ **prosocial**	1.5	0.7	2.1	0.04	0.07	2.96
3. **Mothers' MCS-12 **parent SDQ **hyperactivity**	-1.9	0.9	-2.2	0.03	-3.76	-0.18
4. **Mothers' MCS-12 **parent SDQ **conduct**^1^	-8.8	3.5	-2.5	0.01	-15.68	-2.02

General Linear Model *Dependent variable: *Mothers' PCS-12 or MCS-12 score; *Independent: *Mother's age, age and gender of the child. ^1^Due to skewness, the logarithmic value of parent SDQ conduct score was used. SDQ = Strengths and Difficulties Questionnaire.

## Discussion

Results indicate that mothers, but not fathers, who are caregivers of school-age children with AS/HFA are at increased risk of impaired physical well-being. We also found that the impaired maternal HRQL in the AS/HFA group is related to the extent of symptoms of hyperactivity and conduct problems in the child.

Since there is sparse data about the HRQL of parents who are caregivers of children with AS or HFA, we need to attempt to compare our results with the results of studies dealing with the well-being of parents of children with other types of disorders. Hence, our findings that the mothers of children with AS/HFA report impaired physical well-being resembles previous findings on caregivers of children with intellectual disability [13] cerebral palsy [40] and mental disorders [14,15]. For example, Emerson [13] reported that 20 percent of mothers of children with intellectual disability versus three percent of mothers of children without intellectual disability considered themselves to be "physically ill" due to the child's difficulties. Seltzer et al. [14], and Magana et al. [15] also found more physical symptoms or increased rates of physical health problems among mothers of adult children with severe mental disorders. Notably, there are also studies which suggest a genetically-linked increased rate of autoimmune disorders in parents of individuals with PDDs [41].

The present study did not detect statistically significant differences between mothers in the AS/HFA group and mothers of the control group regarding their self-perceived mental well-being. This is in contrast with many previous studies that have shown that mothers' mental health is related to the child's disability [8,12-14,40,42]. Of course it is possible that our failure to match such findings is due to the low power of the current study, given that the relatively small differences in mental health between parents in the AS/HFA and control groups did not reach statistical significance. Nevertheless, could there be any way to explain our findings of relatively good mental, but poor physical well-being among the mothers? Drawing on previous studies, we note that Weiss [7] reported that psychosomatic problems were common manifestations of stress related to caregiving in parents of children with PDDs, and based on their findings, Magana et al. [15] discussed whether mothers of adult children with mental illness were particularly vulnerable to physical health problems. From another standpoint, one might speculate whether the poorer self-rated maternal physical health in the AS/HFA group could be associated with particular personality traits. From a strictly theoretical perspective, a discrepancy between mental and physical health in these mothers could be related to the presence of alexithymic traits, meaning a reduced ability to engage in explicit emotional processing. A relationship between alexithymic personality and somatization has been reported [43,44], and research on adults with AS has also found high rates of alexithymia in these individuals [45]. However, the current study did not determine the presence of alexithymic traits in parents of children with AS/HFA.

Our finding that maternal physical health was poorer than paternal physical health in the AS/HFA group resembles results in a previous report on parenting a child with Down's syndrome, where mothers were more exhausted than fathers [12]. However, our finding, that self-rated mental health did not differ between mothers and fathers of children with AS/HFA, is in contrast with previous studies. To illustrate, other researchers have reported more anxiety [17] exhaustion [12] child-care related stress, pessimism about the child's future, and use of antidepressants in mothers of these children [6].

In similarity with the results by Hastings [16,17], we found that maternal, but not paternal health in the AS/HFA group was related to particular behaviour characteristics of the child. Maternal mental health was related to the extent of symptoms of hyperactivity and conduct problems in the child, and maternal physical and mental health were related to the prosocial behaviour of the child. Previous research has suggested that coexisting behaviour problems in a child could be more stressful for parents than the severity of the child's core disability [16,19]. Thus, our finding that maternal health was related to the extent of general behaviour problems of the child, and not to the degree of autistic symptoms reflected in the ASSQ-score, may be in similarity with findings in other studies. However, regarding the social competence of the child, which is a primary aspect of PDDs, we do note that our SDQ data indicates a relationship between maternal health and the prosocial behaviour of the child (ability to be considerate, to share, to be helpful and to be kind to younger children). Notably, the items and wordings of the ASSQ and of the prosocial behaviour scale of the SDQ cover somewhat different aspects of social competence in children. In consistency with other authors [2], we believe that the prosocial behaviour scale of the SDQ may yield additional useful information about the behaviour characteristics of children with PDDs.

The main strength of the present study is the use of a well-defined sample of 32 school-age children with ICD-10 diagnosed AS or HFA and the control group of typically developing children. Likewise, the use of the SF-12, a well-validated measure of HRQL, and parent as well as teacher-ratings of the children's behaviour, strengthen our report.

However, there are also limitations of the present study, which must be acknowledged. The sample of individuals with AS/HFA was rather small. During the sampling procedure, children with comorbid medical disorders or ongoing medication were excluded from our sample. Whether the sampling method biased parental results in a positive direction is unknown. Thus, considering the issue of low power, it is quite possible that small differences in HRQL between parents in the AS/HFA and control groups were not detected in the current report. More, there were no statistically significant differences with regard to sociodemographic data between parents in the AS/HFA and control groups in this material. However, more mothers in the AS/HFA group were not employed and were lone parents. These important sociodemographic factors need further investigation in larger studies. Finally, the fact that parental health was only measured by the SF-12, and that no physical examination or review of the parents' medical records was performed, are also limiting factors.

To summarize, we found that parenting a child with AS/HFA was associated with impaired HRQL in mothers, but not in fathers, and that impaired maternal HRQL was associated with higher levels of behaviour problems in the child. We conclude that parental HRQL in children with AS/HFA needs further exploration in larger studies. Moreover, studies exploring the issues related to HRQL and sociodemographic circumstances in these parents would be of great interest.

## Authors' contributions

HA was the main principal investigator collecting the data and preparing the manuscript together with J-OL and HS.

J-OL supervised and participated with great impact at all stages of preparation of this manuscript, and advised on the statistical analysis.

HS was co-conceiver of the idea of this study and made substantial contribution to the analysis and interpretation of data and preparation of the manuscript.
